# Supplementary material for: Instructor-Blinded Study of Pharmacy Student Learning When a Flipped Online Classroom Was Implemented during the COVID-19 Pandemic
Source: Pharmacy (Basel). 2022 May 11;10(3):53. doi: 10.3390/pharmacy10030053 (PMC9149915; doi:10.3390/pharmacy10030053)
Supplement: Supplementary file 1 [file pharmacy-10-00053-s001.zip › SupplementaryMaterialS1.pdf]

# WATERLOO | PHARMACY

# PHARM 362 – Advanced Patient Self-Care
# Winter 2020

## Contact Information


| | |
|---|---|
| Course Co-ordinator: | Nardine Nakhla |
| Email: | Nnakhla@uwaterloo.ca |
| Office Hours: | Thursdays 12-1pm |
| Course Instructors: | Cynthia Richard, Brett Barrett |


## Course Description

Advanced Patient Self-Care teaches students to critically evaluate the use of Prescription (Schedule I) products as well as self-care therapeutic options (e.g., Schedule II and III drugs, unscheduled products, Natural Health Products) in various disease states and patient populations. This course is designed to give the pharmacy student a comprehensive understanding of these products as they relate to patient self-medication and minor ailments. This course is also designed to enhance the students' understanding of common ailments and their treatment, both non-prescription and prescription, necessary for contemporary pharmacy practice in the area of self-care and minor ailments. Emphasis will be placed on the responsibility and role of the pharmacist in accurately triaging patients, determining the appropriate use of non-prescription drugs, in self-medication (i.e., ethical responsibilities, knowledge, experience), and in determining appropriateness of Schedule I therapy. Pharmacist-led minor ailment prescribing schemes across Canada will be described and contrasted.

The main course material will be presented as case-based and evidence-based didactic lectures, and student participation in class discussions will be expected. Tutorials will be used for a hands-on application of the concepts discussed in lectures via patient counselling/ role-plays. These weekly case studies and role-plays will provide the opportunity for students to hone their knowledge and skills in non-prescription therapeutics as well as to practice making decisions while conveying reliable and objective information to patients and health-care providers to ensure optimal outcomes from their selected, evidence-based therapy.

There will be an option to participate in a research study being conducted by the course instructor on pharmacy students' confidence in managing and counselling on minor ailments. This study has been reviewed and received ethics clearance through a University of Waterloo Research Ethics Committee (ORE#41753). If you have questions for the Committee contact the Office of Research Ethics at https://uwaterloo.ca/research/office-research-ethics

Requisites
Prerequisite: PHARM 220, PHARM 228, Pharmacy students only

## Learning Objectives*

Upon completion of this course students will be able to:

1. Utilize and adapt the Pharmacists' Patient Care Process to efficiently identify, prevent, and solve drug therapy problems in patients presenting with minor ailments and/or self-care concerns.

| |
|---|
| 2. Generate highly-effective, evidence-based care plans for patients presenting with minor ailments and/or self-care concerns. |
| 3. Effectively justify and defend recommendations for the management of patients with minor ailments and/or self-care concerns both verbally and in writing. |
| 4. Demonstrate appropriate patient counselling skills for nonprescription consults and initiation of prescription therapy for minor ailments. |
| 5. Using knowledge of current trends facing patient self-care, identify how these can be leveraged to support financial sustainability of community pharmacies. |
| 6. Create clinical resources to meet the needs of patients and pharmacists in the context of self-care consults and minor ailment services. |

*See the Appendix for a detailed list of the learning objectives, AFPC outcomes, and NAPRA competencies.

## Student Assessment

| Assessment | Brief Description | Value/100 |
|---|---|---|
| **Professionalism** | Students are expected to demonstrate professionalism in this course and unprofessional behaviour will result in a deduction from your final mark, at the discretion of the instructor. | Up to 5% deduction |
| **Role-Play** | Students will role-play a self-care scenario with a standardized patient | 15% |
| **Spotlight on Self-Care**: Publication Competition | Students will create a 2-page magazine insert on an assigned topic intended for a healthcare professional audience. The top five submissions will be published in Pharmacy Practice + Business. Students will also be required to translate this information into a patient-friendly handout + a pharmacists' companion sales checklist that can be used in practice. | 20% |
| **Midterm Exam** | MCQ & Short-answer | 30% |
| **Final Exam** | MCQ & Short-answer | 35% |

**Required Reading:**

Compendium of Therapeutics for Minor Ailments. Ottawa, ON: Canadian Pharmacists Association. [Available online: https://myrxtx.ca/search]

Enhancing Eye Care through Interprofessional Collaboration course. [Available online: https://uwaterloo.ca/pharmacy/degrees-and-professional-development/distance-education-continuing-professional-development/enhancing-eye-care-through-interprofessional-collaboration ]

- Note: Students are entitled to a discounted price of $5/participant (course coordinator to provide a promo code].

**Supplemental Reading:**

Krinsky DL, Berardi RR, Ferrari SP, et.al. Handbook of Nonprescription Drugs: An Interactive Approach to Self-Care. 19th ed. Washington, DC: American Pharmaceutical Association; 2018.

Herrier R, Apgar D, Boyce R, Foster S. Patient Assessment in Pharmacy. China: McGraw-Hill Education; 2015. [Available through AccessPharmacy]

University of Saskatchewan- Guidelines for Minor Ailment Prescribing: https://medsask.usask.ca/professional-practice/minor-ailment-guidelines.php

iClicker® will not be used in this course.

Top Hat® will be used in this course.

A passing grade for this course is 60%. Assignments that are not submitted on time will have 20% of the assignment grade deducted per day that it is late.

A supplemental exam will not be offered to students who do not achieve a passing grade in this course.

Group work accounts for 0% of the total mark.

## Territorial Acknowledgement

We acknowledge that the University of Waterloo is located on the traditional territory of the Neutral, Anishnaabeg and Haudenosaunee people.

## Course Topics

A complete list of activities is available on LEARN or on other online learning platforms. Assignment details will be posted on LEARN or on other online learning platforms.

## Student Evaluation

In this course, there are activities that are graded, assignments that are mandatory but not graded or events for which the students' presence is mandatory. Students are reminded that they should view these activities with the same professionalism as they would a job. Attendance and participation is expected and non-attendance, lack of participation or non-completion of assignments will affect an individual student's grade in this course.

A student will be excused for a mandatory activity only under exceptional and unforeseen circumstances. While it is not possible to list all such circumstances, examples include: death of an immediate family member; significant illness of a family member; serious personal illness or injury requiring medical attention. Please note that student travel plans are not considered acceptable grounds for granting an alternative examination time or altering assignment deadlines. Questions should be directed to the Associate Director, Curriculum, Dr. Cynthia Richard.

When students believe their illness will impact their academic studies in a course, they must contact the course coordinator for that course before or within 24 hours of a mandatory activity and submit a completed Verification of Illness (VIF) Form (available online: https://uwaterloo.ca/campus-wellness/sites/ca.campus-wellness/files/uploads/files/VIF-online.pdf) to the Pharmacy Undergraduate Office. Students whose illness delays the submission of the VIF should communicate via email or phone to avoid delays or difficulties with accommodation decisions. When a VIF is registered all course coordinators will be notified by email.

In the event that a student unavoidably misses a mandatory activity, a make-up activity may be scheduled for that student at the sole discretion of the course coordinator. In the case of a missed exam, a new exam may be administered. This new exam may be the same as the original exam, may be a different exam, may be of a different format (e.g. an oral test), or may be of a different mix of questions (e.g. the percentage of specific topics on the new exam may differ from the original). In any case, the final decision of the exam format and date that it will be administered will rest solely with the course coordinator.

Students must discuss the VIF-specified degree of incapacitation with their course coordinators so that their actions are not misinterpreted or viewed with suspicion. A "severe" incapacitation indicates students are unable to engage in any academic studies during the period indicated. **If they attend classes/labs, submit assignments/reports or write tests/exams during this period, they are at risk of being accused of "misrepresentation"; an academic offence, which carries a severe penalty that includes suspension.** A "moderate" incapacitation could trigger a similar allegation, if students claim to be well enough to participate in some tests, while too ill to participate in others. Thus, in these cases it is important that students discuss their degree of incapacitation with their course coordinators so that their actions appear credible.

More information regarding VIFs can be found on the Faculty of Science's website: https://uwaterloo.ca/science/current-undergraduate-students/frequently-asked-questions and the Undergraduate Calendar: http://ugradcalendar.uwaterloo.ca/page/Regulations-Accommodations.

Students may receive a grade of Incomplete (INC) in a course where the student has been unable to complete course work because of verifiable illness or extenuating circumstances.  These students must contact the course coordinator to formally request an INC for the course. Course coordinators will clearly define the outstanding course element(s) to be completed and will specify the final date when all outstanding course element(s) are to be received for grading. The onus is on the student to complete all outstanding course elements(s). An online INC Grade Form will then be submitted by the course coordinator no later than the grade submission deadline for the course. Failure to complete work by the deadline indicated will result in the INC grade being changed to FTC (failure to complete).  The course will be weighted as a grade of 32 for purposes of calculating the student's average.

Students that wish to have an assignment or exam re-graded must submit a written request that details why it should be re-graded. This written request must be delivered to the course coordinator within one month (see Appendix B of Policy #70) of the date that the grade was available, and must contain supporting evidence (e.g. from literature, textbooks, or other sources). Students should note that grades may stay the same, be increased or be decreased after re-grading.  Depending upon the request, the course coordinator may decide to re-grade the whole assignment or test, and not just the question that is being challenged. Students should not mark in any way assignments or tests that they think may warrant a re-examination. Only assignments and tests that are completed in indelible ink are eligible for re-grading.

In accordance with University policy, all assessments will be retained for one year after term grades become official. After that time, they will be destroyed in compliance with the University's confidential shredding procedures.

Students will be given one opportunity, for a maximum time to be specified by the instructor, to review their midterm papers after they have been graded.  Students will be given one opportunity, for a maximum time to be specified by the instructor, to review their final exam after course grades have been released, if they submit a written request to the course coordinator within a 6-month period. Requests to see exams for a second time will not be accommodated.  When viewing graded exams, students are not permitted to take notes, or have any electronic devices on their person.

## Changes to Course Outline

The topics addressed in this course, as well as the schedule of topics, may be revised as the course progresses. Any changes will be announced in class and posted on LEARN or on other online learning platforms. **The assessment weights CANNOT be altered.** There is one exception. When a student has been excused from completing a mandatory assessment (a midterm, for example), the decision about how and when this assessment will be completed is at the discretion of the course coordinator. In this instance, the coordinator may choose to alter assessment weights by re-allocating marks from a midterm to a final exam. See the Student Assessment section of this document for more information.

## Intellectual Property

Students should be aware that this course contains the intellectual property of their course coordinators, TA, and/or the University of Waterloo.  Intellectual property includes items such as:

- Lecture content, spoken and written (and any audio/video recording thereof);
- Lecture handouts, presentations, and other materials prepared for the course (e.g., PowerPoint slides);

- Questions or solution sets from various types of assessments (e.g., assignments, quizzes, tests, final exams); and
- Work protected by copyright (e.g., any work authored by the course coordinator or TA or used by the course coordinator or TA with permission of the copyright owner).

Course materials and the intellectual property contained therein, are used to enhance a student's educational experience. However, sharing this intellectual property without the intellectual property owner's permission is a violation of intellectual property rights. For this reason, it is necessary to ask the course coordinator, TA and/or the University of Waterloo for permission before uploading and sharing the intellectual property of others online (e.g., to an online repository).

Permission from a course coordinator, TA or the University is also necessary before sharing the intellectual property of others from completed courses with students taking the same/similar courses in subsequent terms/years. Doing so without expressed permission is considered a violation of intellectual property rights.

Please alert the course coordinator if you become aware of intellectual property belonging to others (past or present) circulating, either through the student body or online. The intellectual property rights owner deserves to know (and may have already given their consent).

## Students with Disabilities

AccessAbility Services (https://uwaterloo.ca/accessability-services/), located in Needles Hall, room 1401, collaborates with all academic departments to arrange appropriate accommodations for students with disabilities without compromising the academic integrity of the curriculum. If you require academic accommodations to lessen the impact of your disability, please register with AccessAbility Services at the beginning of each academic term.

## Expectation of Academic Integrity

**Academic Integrity**

In order to maintain a culture of academic integrity, members of the University of Waterloo community are expected to promote honesty, trust, fairness, respect and responsibility. Please refer to: http://www.uwaterloo.ca/academicintegrity/Students/index.html.

The Office of Academic Integrity has developed a tutorial that helps students recognize and avoid academic integrity offences https://uwaterloo.ca/academic-integrity/basic-info).

Using another student's Top Hat® account or bringing another student's iClicker® to class to help them receive participation marks is a breach of academic integrity. If you do so, all students involved will be subject to penalty. It is a breach of academic integrity to sign into a Seminar course for a classmate.

Text matching software (Turnitin®) may be used to screen assignments in this course. Turnitin® is to verify that all materials and sources in assignments are documented. Students' submissions are stored on a U.S. server, therefore students must be given an alternative (e.g., scaffolded assignment or annotated bibliography), if they are concerned about their privacy and/or security. Students will be given due notice, in the first week of the term and/or at the time assignment details are provided, about arrangements and alternatives for the use of Turnitin® in this course.

Students are reminded that in accordance with University *Policy 71 – Student Discipline*, the "*obtaining, distributing, or receiving of any confidential academic material without the express consent of the instructor*" is considered an academic offence. As such, students should not procure, use, attempt to use or distribute any

improper or unauthorized materials related to tests, including, but not limited to: test questions, copies of paper tests, test files of any type (electronic or non-electronic) and/or test passwords. Any intentional attempt to access the test outside of the scheduled test time, and/or disable or tamper with the test in any form, including, but not limited to: paper tests/ExamSoft/SofTest/Examplify's security features may be subject to the University's academic discipline procedure.

**Discipline**

Students are expected to know what constitutes academic integrity, to avoid committing academic offenses, and to take responsibility for their actions. Students who are unsure whether an action constitutes an offense, or who need help in learning how to avoid offenses (e.g., plagiarism, cheating) or about 'rules' for group work/collaboration should seek guidance from the course coordinator, academic advisor, or the Associate Dean of Science for Undergraduate Studies. For information on categories of offenses and types of penalties, students should refer to Policy #71, Student Discipline http://www.adm.uwaterloo.ca/infosec/Policies/policy71.htm. For information on typical penalties, students should check Guidelines for the Assessment of Penalties https://uwaterloo.ca/secretariat/policies-procedures-guidelines/guidelines/guidelines-assessment-penalties.

**Appeals**

A decision or penalty imposed under Policy #33 (Ethical Behaviour), Policy #70 (Student Petitions and Grievances) or Policy #71 (Student Discipline) may be appealed, if there is a ground. Students, who believe they have a ground for an appeal, should refer to Policy #72 (Student Appeals) http://www.adm.uwaterloo.ca/infosec/Policies/policy72.htm.

**Student Grievances**

Grievance: A student who believes that a decision affecting some aspect of their university life has been unfair or unreasonable may have grounds for initiating a grievance. Read Policy #70, Student Petitions and Grievances, Section 4. https://uwaterloo.ca/secretariat/policies-procedures-guidelines/policy-70. When in doubt, contact the Director of Admissions, Professional Relations & Undergraduate Affairs who will provide further assistance.

# Appendix:
# Learning Objectives and Associated AFPC Outcomes and NAPRA Competencies

***Upon completion of PHARM 362 students will be able to:***

| | | AFPC Outcomes | NAPRA Competencies |
|---|---|---|---|
| 1 | Utilize and adapt the Pharmacists' Patient Care Process as a systematic approach to minor ailment and/or self-care counselling. This includes identifying, preventing and solving drug-related problems related to minor ailments and/or self-care therapeutic | CP1.2, CP2.1, CP2.2, CP2.3, SC1.2, SC2.4, CP1.1, CP2.4.1, CP2.4.2, CP2.4.5, CP2.4.4c | 2.3, 2.4, 2.5, 2.1, 2.2, 2.6 |
| 2 | Further develop clinical assessment skills to differentiate appropriate self-care candidates from those requiring pharmacist-led minor ailment services and from those requiring referral. | CP1.3, CP2.1, CP2.2, CP2.3, SC1.3, SC2.4, CP1.2, CP1.5, CP2.4.4c | 2.3, 2.4, 2.5, 8.4 |
| 3 | Apply principles of evidence-based medicine to the selection and justification of primary and secondary pharmacologic or nonpharmacologic therapeutic options for minor ailments. | CP1.1, CP2.2, SC1.1, SC2.4, SC2.3, SC2.1 | 2.5, 6.1, 6.2, 6.3 |
| 4 | Demonstrate appropriate patient counselling. This includes discussing self-care strategies and proper use of nonprescription medications in special populations, such as paediatric, geriatric, and pregnant patients. | CP1.4, CP2.4.1, CP2.4.3, CP2.4.5, CM1.1, CM1.2, CM1.3, CM1.4, CM1.5, CM1.6, CM1.7 | 5.1, 7.1, 7.2, 7.3 |
| 5 | Explain current trends facing patient self-care and how they may translate into opportunities for pharmacy. | CM1.3, CM1.5, SC4.1 | 1.1, 1.2, 1.3, 6.3, 9.2 |
