# Supplementary material for: Instructor-Blinded Study of Pharmacy Student Learning When a Flipped Online Classroom Was Implemented during the COVID-19 Pandemic
Source: Pharmacy (Basel). 2022 May 11;10(3):53. doi: 10.3390/pharmacy10030053 (PMC9149915; doi:10.3390/pharmacy10030053)
Supplement: Supplementary file 1 [file pharmacy-10-00053-s001.zip › SupplementaryMaterialS3.pdf]

Evaluation of the Impact of a Flipped Classroom on Pharmacy Students' Knowledge & Confidence in Advanced Self-Care and Minor Ailment Management

Knowledge Assessment Questions

THESE QUESTIONS ARE AIMED TO ASSESS STUDENT FACTUAL KNOWLEDGE ON SELF-CARE AND MINOR AILMENT TOPICS. THE QUESTIONS ARE BLINDED TO THE COURSE INSTRUCTOR AND WILL FOLLOW THE SELF-CONFIDENCE QUESTIONS IN THE PRE- AND POST-COURSE ASSESSMENT SURVEY.

1. What is the most appropriate recommendation regarding breastfeeding for HIV-infected mothers?

A. Do not breastfeed your infant
B. You may practice breastfeeding if the viral load in your blood is undetectable
C. You may breastfeed freely as there is no risk of HIV transmission through breast milk

2. To what extent can you recommend calorie restriction to achieve weight loss without close medical supervision?

A. Limit caloric intake to 1800 kcal/day
B. Limit caloric intake to 1500 kcal/day
C. Limit caloric intake to 1200 kcal/day
D. Limit caloric intake to 800 kcal/day
E. Limit caloric intake to 400 kcal/day

3. Which minor ailment typically presents with an acidic pH in the vagina?

A. Vulvovaginal candidiasis
B. Atrophy
C. Bacterial vaginosis
D. Trichomoniasis

4. Which Non-Steroidal Anti-Inflammatory drug (NSAID) poses the lowest risk for gastrointestinal adverse events, such as ulcers or gastrointestinal bleeding?

A. Diclofenac
B. Naproxen
C. Ibuprofen
D. Indomethacin
E. Celecoxib

5. Which Non-Steroidal Anti-Inflammatory drug (NSAID) poses the lowest risk for cardiovascular adverse events, such as myocardial infarction and stroke?

A. Diclofenac

B. Naproxen
C. Ibuprofen
D. Indomethacin
E. Celecoxib

6. When treating atopic dermatitis, how long is an appropriate trial for a topical corticosteroid before an alternative agent should be considered?

A. Two weeks
B. One month
C. Three months
D. Six months

7. Which of the following would NOT be classified as a "red flag" warranting referral for assessment by a physician in the context of low back pain?

A. Immunosuppression
B. Bladder dysfunction
C. Persistent leg weakness
D. Age < 30 years

8. Which of the following options is a <u>recommended treatment</u> for constipation-predominant irritable bowel syndrome?

A. Align probiotics
B. Citalopram
C. Amitriptyline
D. Lactulose

9. Which of the following non-prescription products is a <u>first line agent</u> for treating nausea and vomiting in the first 6 weeks of pregnancy?

A. Dimenhydrinate tablets
B. TUMS chewable tablets
C. Vitamin B6 tablets
D. Gravol ginger tablets

10. Which of the following types of infant formulas is the MOST appropriate for an infant with an allergy to cow's milk?

A. Soy-based
B. Extensively hydrolyzed
C. Partially hydrolyzed
D. Lactose free

11. Which of the following agents may be used to treat both Bacterial Vaginosis AND Trichomoniasis?

A. Metronidazole oral capsules
B. Metronidazole topical gel
C. Clindamycin topical cream
D. All of the above

12. What is the first line treatment for a painful external hordeolum that appeared 24 hours ago?

A. Warm compresses
B. Steroid injections
C. Topical antibiotics
D. Oral antibiotics

13. When the luteinizing hormone surge is detected by an ovulation test or fertility kit, ovulation will likely occur in the next:

A. 6-12 hours
B. 12-48 hours
C. 48-72 hours
D. 5 days

14. Which of the following non-prescription products <u>does not</u> have restrictions for use in the elderly?

A. Diphenhydramine
B. Methocarbamol
C. Dimenhydrinate
D. Bisacodyl

15. Which of the following products is NOT effective as an analgesic in pharyngitis?

A. Peridex
B. Tantum
C. Cepacol
D. Chloraseptic

16. Which patient is <u>most likely</u> to be presenting with bacterial pharyngitis?

A. A six-year-old female complains of sore throat but no cough and no fever
B. A fifty-year-old male complains of sore throat with cough and fever
C. A sixteen-year-old male complains of sore throat with cough and fever
D. A fifty-year-old female complains of sore throat with fever and rhinitis but no cough

17. Which of the following is NOT a typical symptom of cystitis?

A. Fever

B. Urinary urgency
C. Dysuria
D. Suprapubic pain

18. When using a rapid antigen detection test (RADT) to test for bacterial pharyngitis in a child, which results require confirmation with culture?

A. Positive results
B. Negative results
C. Both positive and negative results
D. None

19. In sports-related injuries, which symptom DOES NOT require immediate referral for medical attention?

A. Headache
B. Joint swelling
C. Blurred vision
D. Nausea

20. Which of the following topical antibacterials is NOT a first line agent for treating superficial, mildly infected wounds?

A. Fucidin (fusidic acid)
B. Bactroban (mupirocin)
C. Flamazine (silver sulfadiazine)
D. Polysporin (bacitracin and polymixin B)

Unique Identifier

Please enter the LAST FOUR digits of your student number as a unique identifier

e.g., 2561; this identifier will be used to correlate your pre- and post-course results

______
